# Parental effects of physiological integration on growth of a clonal herb

**DOI:** 10.3389/fpls.2024.1518400

**Published:** 2025-01-17

**Authors:** Li-Min Zhang, Li-Li Zheng, Fei-Hai Yu

**Affiliations:** Institute of Wetland Ecology & Clone Ecology, Taizhou University, Taizhou, Zhejiang, China

**Keywords:** clonal integration, clonal plant, genotype, severance, transgenerational effects

## Abstract

Although numerous studies have independently tested the roles of physiological integration and parental effects on the performance of clonal plant species, few have assessed them simultaneously. Moreover, the capacity for physiological integration differs greatly within species of clonal plants. We conducted a greenhouse experiment with eight genotypes of the clonal herb *Hydrocotyle verticillata.* In the first phase, we either severed or maintained the connections between the original proximal nodes (the basal portion) and the new distal nodes (the apical portion) of each genotype. In the second phase, the ramets in the apical portion produced in the first phase were selected and cultivated, and their connections were subjected to the same severance treatments. In the first phase, the negative effects of severance on the apical portion balanced the positive effects of severance on the basal portion, resulting in no net effect of severance on total mass, leaf mass, stem mass, and ramet number for the whole clone. In the second phase, the effects of parental severance on stem mass of the apical portion of *H. verticillata* varied among the eight genotypes. Additionally, the positive effect of physiological integration on offspring generations was greater in the apical portion and the whole clone of one genotype when the parental connections were intact than when they were severed, whereas it was greater in the apical portion of another genotype when the parental connections were severed than when they were intact. Our results suggest that clonal parental effects can influence the capacity for physiological integration of offspring generations and that these effects may differ among genotypes within a species.

## 1 Introduction

The environmental conditions experienced by parents can influence the phenotype and performance of their offspring, a phenomenon known as parental or transgenerational effects (Agrawal, 2001; Latzel et al., 2010; Pigeon et al., 2019; Lukic et al., 2023). Parental effects play crucial roles in population dynamics and evolution, as they can induce substantial heritable variation and thus impact the ability to adapt to rapidly changing environments (Badyaev and Uller, 2009; Latzel and Klimešová, 2010; Münzbergová et al., 2019). These effects have been widely demonstrated in both clonal and non-clonal plants (Baker et al., 2019; Dong et al., 2019; Veselá et al., 2021; Xue et al., 2022; Latzel et al., 2023). Compared with non-clonal plants (i.e., sexually reproducing plants), parental effects are especially important for clonal plants because asexual reproduction circumvents meiosis, which is associated with resetting epigenetic memory (Paszkowski and Grossniklaus, 2011; Verhoeven and Preite, 2014; Wang et al., 2020). Additionally, clonal plants are often dominant in many ecosystems; therefore, their ability to adapt to environmental variation likely affects not only their own populations but also the functioning of communities and even whole ecosystems (Ma et al., 2023; Wang et al., 2024).

Increasing evidence has shown that stressful environments (e.g., drought, herbivory, shading) induce parental effects that increase offspring fitness and thereby adapt when offspring establish in environments similar to those of their parents (Herman and Sultan, 2011; Holeski et al., 2012; Ballhorn et al., 2016; Baker et al., 2018). These adaptive phenomena have also been reported in some clonal species (Dong et al., 2017; González et al., 2017; Li et al., 2018). In addition, clonal fragments produced in favorable parental environments benefit the subsequent growth of clonal offspring, as the provisioning of resources can directly influence the initial status of vegetative propagules (Dong et al., 2018; Portela et al., 2020). For example, González et al. (2017) reported that parent ramets of *Trifolium repens* grown under better conditions produced larger vegetative propagules that enabled offspring to grow better. Vegetative propagules of clonal plants are larger in size and mass than sexual propagules, giving them a substantial advantage in terms of resource provisioning (Dong et al., 2019).

Clonal plants, which are capable of clonal growth or vegetative propagation, are widespread in nature (de Kroon and van Groenendael, 1997). The distinguishing feature of clonal plants is their capacity for physiological integration, i.e., the translocation of nutrients, water, carbohydrates, and signals between connected ramets of the same clone (Alpert, 1996; Xu et al., 2010; Touchette et al., 2013; Wang et al., 2017; Guan et al., 2023). A large body of evidence shows that physiological integration can increase the survival, growth, and reproduction of ramets in diverse habitats (Yu et al., 2008; Roiloa and Retuerto, 2012; Dong et al., 2015; Gruntman et al., 2017; Adomako et al., 2020; Estrada et al., 2020; Wang et al., 2021a). These positive effects of physiological integration can increase the competitive ability of clonal plants (Wang et al., 2016, 2021b) and make important contributions to the invasiveness of alien clonal plants (Elgersma et al., 2015; Wang et al., 2017; Chen et al., 2019). Therefore, physiological integration has ecological advantages and is likely a major reason why clonal plants are often dominant in many ecosystems (Song et al., 2013).

Numerous studies have independently tested the roles of physiological integration and parental effects on the performance of clonal plant species (Latzel and Klimešová, 2010; Elgersma et al., 2015; Lu et al., 2015; González et al., 2017; Xing et al., 2024). To date, however, only one study has assessed them simultaneously, showing that physiological integration could influence the performance of the aquatic clonal plant *Pistia stratiotes* across generations (Adomako et al., 2021). Furthermore, the capacity for physiological integration differs greatly within species of clonal plants (Alpert et al., 2003; Si et al., 2020). Therefore, genotypes with a higher capacity for physiological integration may produce larger offspring ramets and thus benefit more on their subsequent growth than those with a lower capacity for physiological integration. Nevertheless, no study has tested whether the parental effect of physiological integration on offspring performance varies between genotypes of the same species.

Therefore, we conducted a greenhouse experiment with eight genotypes of the clonal herb *Hydrocotyle verticillata.* In the first phase, connections between the original proximal nodes (the basal portion) and the new distal nodes (the apical portion) of eight *H. verticillata* genotypes were either severed or kept intact. In the second phase, the ramets in the apical portion produced in the first phase were selected and cultivated, after which their connections were subjected to the same severance treatments. As both physiological integration and parental effects have the potential to influence plant growth (Song et al., 2013; Donelan and Trussell, 2015; Estrada et al., 2020), we tested the following hypotheses: (1) parental severance affects the offspring performance of *H. verticillata*; (2) parental severance on offspring performance may differ between the genotypes because different genotypes may vary in their capacity for physiological integration; and (3) the positive effect of physiological integration on offspring growth may be stronger when their parental connections are intact than when they are severed.

## 2 Materials and methods

### 2.1 Study species and collection of genotypes

In this study, we used a perennial clonal plant *Hydrocotyle verticillata* Thunb. [Araliaceae; previously misidentified as *Hydrocotyle vulgaris* in China (Liu et al., 2017; Wang et al., 2020; Zhang et al., 2022a)]. The species is native to North and South America and has been widely introduced to various regions of the world (Kozeko et al., 2024). *H. verticillata* has been introduced to China for over 30 years. Typically, this species is capable of producing ramets with a petiolate leaf and filamentous roots at each node of a horizontal creeping stem (Winkel and Borum, 2009). *H. verticillata* is considered potentially invasive because of its rapid vegetative reproduction and high phenotypic plasticity. This species can thrive in a wide range of habitats, including terrestrial, waterlogged, and fully submerged environments (Lim et al., 2014). Furthermore, genotypes of *H. verticillata* can differ in the capacity for physiological integration (Si et al., 2020).

The initial ramets of *H. verticillata* were collected from five provinces in southern China in 2016 and taken to a greenhouse at Taizhou University in Taizhou, Zhejiang Province, for vegetative propagation. The genotypes of these ramets were determined through amplified fragment length polymorphism (AFLP) based on genomic DNA (Wang et al., 2020). On 6 July 2022, eight genotypes (labeled A, B, C, D, E, F, G, and H) were selected and vegetatively propagated in containers filled with a 1:1 (v/v) mixture of potting soil and sand in the greenhouse at Taizhou University. Forty stem fragments per genotype, each consisting of one node plus 1.5 cm of each of the two adjacent stem internodes, were individually planted in pots (10 cm diameter × 8 cm height) filled with the same soil mixture. After one month, each node produced a new stem with 3–5 nodes, and the second node (from the apex), along with its two connected internodes were cut off to ensure that all these isolated nodes were in the same ontogenetic stage. For each genotype, 26 similar-sized ramets were selected, 10 of which were used for measuring initial dry biomass (genotype A: 11.4 ± 0.5 mg; B: 10.4 ± 0.4 mg; C: 10.3 ± 0.3 mg; D: 12.1 ± 0.9 mg; E: 10.7 ± 0.5 mg; F: 11.9 ± 0.5 mg; G: 11.5 ± 0.5 mg; H: 10.9 ± 0.5 mg; ANOVA results: *F*_7, 72_ = 1.7, *P* = 0.1), and the remaining 16 were used for the experiment described below.

### 2.2 Experimental design

The experiment consisted of two phases. The first phase involved the combination of eight levels of genotype (A, B, C, D, E, F, G, and H) with two severance treatments (severed and intact). Each of the 16 combinations was replicated eight times. One ramet of each genotype was planted at the center of a pot (13 cm in diameter and 11.5 cm in height) filled with equal volumes of river sand and potting soil. The soil mixture contained 1.65 g kg^−1^ total nitrogen and 0.15 g kg^−1^ total phosphorus. After three weeks, all initial (parental) ramets produced a new creeping stem, at least 10 cm long with one node plus an apex, and were rooted in the same separate pot. The original proximal nodes were labeled as the basal portion, whereas the new distal nodes were termed as the apical portion. In the severed treatment, the stem connecting the basal and apical portions was cut halfway between them. In the intact treatment, the portions remained connected. The first phase of the experiment began on 5 August 2022, and severance occurred on 26 August. The treatments were continued for 7 weeks and ended on 14 October 2022.

The second phase of the experiment began on 14 October 2022. At the end of the first phase, three randomly chosen replicates were harvested to measure growth traits. The remaining five replicates were used in the second phase. Two ramets (specifically, the second and third younger ramets along the main stem) were chosen from the apical portion of the five replicates, and thus, ten ramets of each type were obtained. The offspring ramets derived from each type of the parent plant were assigned to the same two severance treatments as described in the first phase. In the second phase, the experiment used a fully factorial design with five replicates of 32 treatment combinations, with the eight genotypes crossed by two parental severance treatments (severed and intact) crossed by two offspring severance treatments (severed and intact). Treatments in the second phase also continued for 7 weeks, ending on 23 December 2022.

The pots were placed on a bench in the greenhouse at Taizhou University. The mean air temperature was 23.2°C, and the humidity was 79.3% in the greenhouse during the experiment, as measured using Hygrochron temperature loggers (iButton DS1923; Maxim Integrated Products, USA). Photosynthetic photon flux density at noon in the greenhouse was 496-1015 µmol m^-2^ s^-1^, as measured weekly with a quantum sensor (LI-250A; LI-COR Biosciences, USA). There were about 12 h of daylight and 12 h of darkness in the greenhouse during the experiment. Tap water was added to each pot every three days to keep the soil water content at 40 – 45%. Soil water content was measured every three days with a soil water probe (Procheck, Decagon Devices, Inc.).

### 2.3 Harvest and measurements

At the end of the first phase of the experiment, three replicates of the plants, which were not used in the second phase, were harvested separately for the basal and apical portions. After we counted the ramet number, the plants were separated into leaves, stems and roots, dried at 70°C for 48 h, and weighed to obtain their biomass. Biomass per ramet (i.e., final total dry mass/number of ramets) was calculated. At the end of the second phase of the experiment, we harvested all five replicates as in the first phase of the experiment. Plants in five pots (one genotype H that was intact in the first phase and severed in the second phase; one genotype F that was severed in both phases; one genotype D that was intact in both phases; and genotypes D and E, which were severed in the first phase and intact in the second phase) were damaged by herbivores during the experiment and excluded from harvest and analysis.

### 2.4 Data analysis

For the first phase of the experiment, two-way ANOVAs were used to test the effects of severance and genotype on ramet number, total mass, leaf mass, stem mass, root mass, and biomass per ramet in the apical portion, the basal portion, and the whole clone. Ramet number, total mass, leaf mass, stem mass, and root mass of the basal portion were ln-transformed to eliminate heteroscedasticity and increase normality. For the second phase of the experiment, we employed three-way ANOVAs to test the effects of parental severance level, offspring severance level, genotype, and their interactions on biomass and ramet number of *H. verticillata.* Before analysis, all the data in the second phase were ln-transformed to increase normality. Statistical analyses were carried out with SPSS 22.0 (IBM Corp., Armonk, New York, USA).

## 3 Results

### 3.1 Effects of genotype and severance on plant performance in the first phase

**Figure 1:**
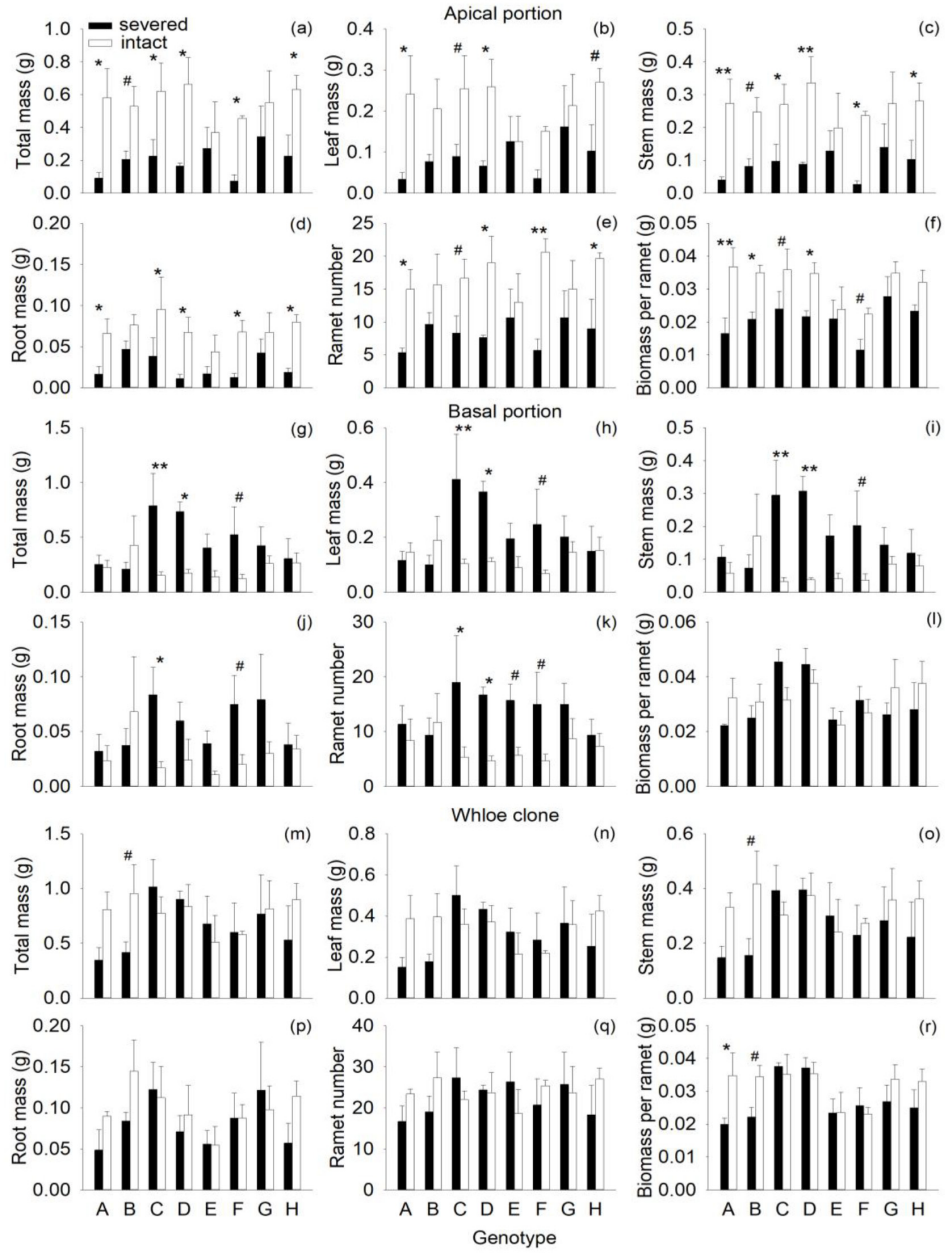
Effects of genotype and severance in the first phase on total mass, leaf mass, stem mass, root mass, ramet number, and biomass per ramet in the apical portion **(A-F)**, the basal portion **(J-L)**, and the whole clone **(M-R)** of *H. verticillata.* Symbols indicate the levels of differences between severed and intact offspring within a genotype (^**^ *P* < 0.01, ^*^ *P* < 0.05, ^#^0.05 < *P* < 0.1.

Severance had highly significant negative effects on all components of mass and on ramet number of the apical portion (**Figures 1A–F**; **Table 1A**: *F* = 19.03 – 35.04; *P* = 0.000 – 0.000). Compared with the intact treatment, severance decreased total mass, leaf mass, root mass, stem mass, and ramet number of the apical portion by 48–65% across genotypes (**Figures 1A–E**). With respect to individual genotypes, severance significantly reduced total mass, stem mass, root mass of genotypes A, C, D, F, and H but had little effect on the apical portion of the other three genotypes (B, E, and G) (**Figures 1A, C, D**). Severance also significantly decreased biomass per ramet in the apical portion of the three genotypes (A, B and D) but had no significant effect on the other five genotypes (C, E, F, G, H). These results suggest that the eight genotypes in the apical portion differed in their response to severance.

**Table 1:** Statistical analysis of the effects of genotype and severance level in the first phase on total mass, stem mass, root mass, leaf mass, ramet number, and biomass per ramet of the apical portion, the basal portion, and the whole clone of *H. verticillata*.

Effect	*df*	Total mass	Stem mass	Root mass	Leaf mass	Ramet number	Biomass per ramet
(A) Apical portion							
Genotype (G)	7, 32	0.49^ns^	0.41^ns^	1.04^ns^	0.61^ns^	0.28^ns^	2.25*^#^*
Severance (S)	1, 32	29.51^***^	35.04^***^	27.54^***^	19.03^**^	27.27^***^	26.17^***^
G × S	7, 32	0.57^ns^	0.46^ns^	0.39^ns^	0.74^ns^	0.80^ns^	0.70^ns^
(B) Basal portion							
Genotype (G)	7, 32	0.52^ns^	0.34^ns^	0.44^ns^	0.83^ns^	0.17^ns^	1.92*^#^*
Severance (S)	1, 32	10.51^**^	16.19^***^	9.96^**^	4.97^*^	16.05^***^	0.12^ns^
G × S	7, 32	1.68^ns^	1.77^ns^	0.80^ns^	1.86^ns^	1.07^ns^	1.13^ns^
(C) Whole clone							
Genotype (G)	7, 32	0.68^ns^	0.59^ns^	1.17^ns^	0.82^ns^	0.17^ns^	2.40^*^
Severance (S)	1, 32	1.12^ns^	2.13^ns^	1.53^ns^	0.34^ns^	0.37^ns^	4.00*^#^*
G × S	7, 32	0.95^ns^	0.91^ns^	0.60^ns^	1.06^ns^	0.74^ns^	1.27^ns^

The F values, degrees of freedom (*df*) and significance levels (^***^ *P*<0.001, ^**^ *P*<0.01, ^*^ *P*<0.05, ^#^0.05<*P*<0.1, and ^ns^ *P*≥0.1) of two-way ANOVA are given. Values are bold where *P <*0.05.

Across genotypes, severance had significant positive effects on total mass, stem mass, leaf mass, root mass, and ramet number of the basal portion (**Figures 1G–K**; **Table 1B**: *F* = 4.97 – 16.19; *P* = 0.000 – 0.033). Considering individual genotypes, severance significantly increased total mass, leaf mass, stem mass, and ramet number of genotypes C and D but had little effect on the other six genotypes (A, B, E, F, G and H) in the basal portion (**Figures 1G–I, K**). Severance had no effect on biomass per ramet of the basal portion in the eight genotypes (**Figure 1L**; **Table 1B**).

The negative effects of severance on the apical portion balanced the positive effects of severance on the basal portion; therefore, severance had no overall effect on total mass, leaf mass, stem mass, and ramet number of the whole clone (**Figures 1M-Q**; **Table 1C**). Genotype significantly affected biomass per ramet of the whole clone (**Figure 1R**; **Table 1C**: *F* = 2.40; *P* = 0.043). There were no significant interaction effects of severance and genotype on biomass or ramet number of the whole clone (**Figures 1M–R**; **Table 1C**).

### 3.2 Effects of genotype, parental severance, and offspring severance on offspring performance in the second phase

**Figure 2:**
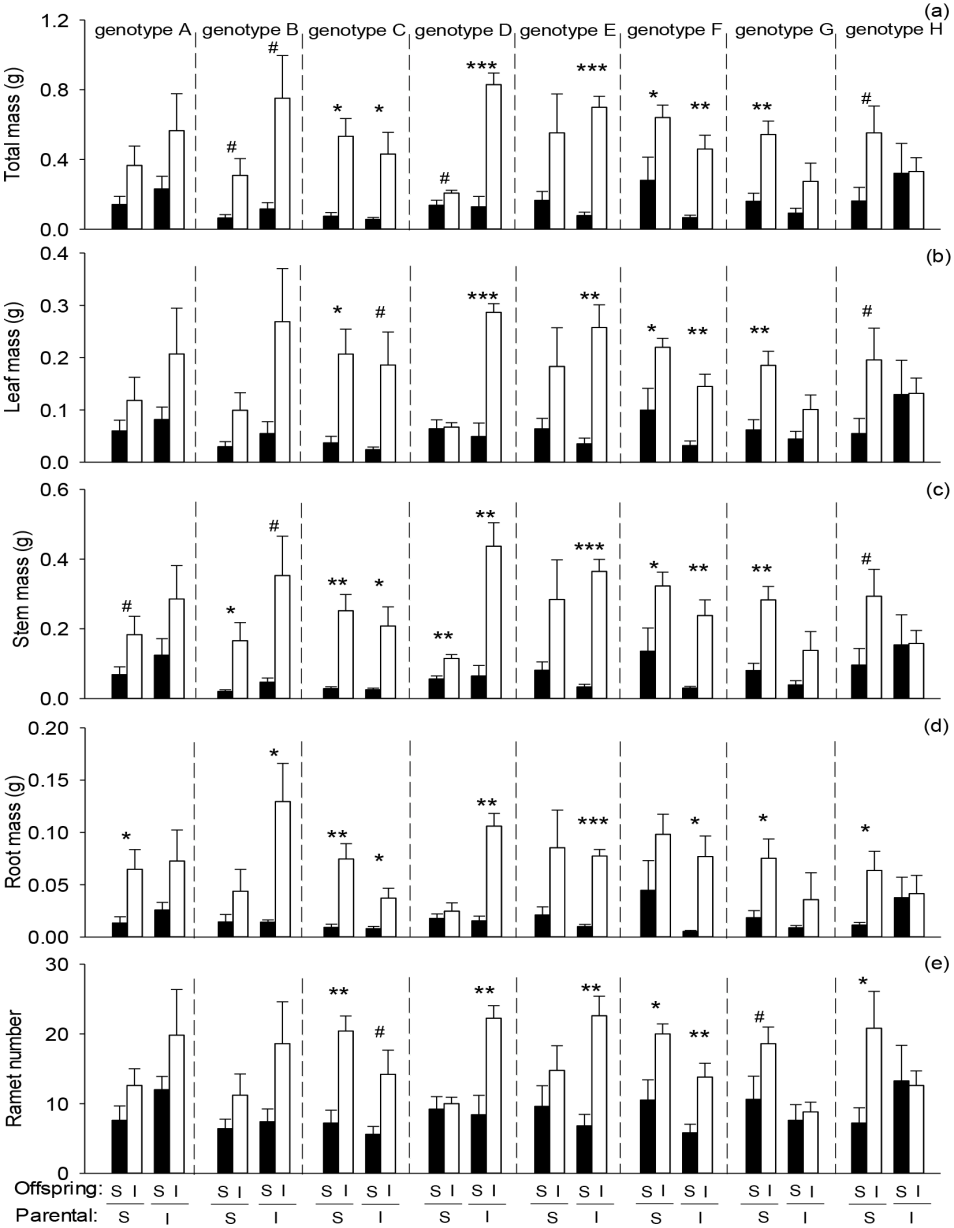
Effects of genotype, parental severance level, and offspring severance level on final **(A)** total mass, **(B)** leaf mass, **(C)** stem mass, **(D)** root mass, and **(E)** ramet number of the apical portion of *Hydrocotyle verticillata* in the second phase. S and I stand for severance and intact, respectively. Symbols indicate the levels of differences between offspring severed and intact within a genotype (***P < 0.001, **P < 0.01, *P < 0.05, and ^#^0.05 < *P* < 0.1).

Offspring severance significantly reduced total mass, leaf mass, root mass, stem mass, and ramet number of the apical portion in the second phase (**Figures 2A–E**; **Table 2**: *F* = 47.91 – 116.93; *P* = 0.000 – 0.000). Parental severance had no significant effect on offspring performance in the apical portion (**Figures 2A–E**; **Table 2**). However, the effects of parental severance on stem mass of the apical portion of *H. verticillata* varied among genotypes in the second phase, as indicated by the significant interactive effect of parental severance × genotype (**Figure 2C**; **Table 2**). Compared with the parental intact treatment, parental severance decreased offspring stem mass of the apical portion in genotypes A, B, D, and E but increased stem mass in genotypes C, F, G, and H (**Figure 2C**). The positive effect of physiological integration on offspring growth was greater in the apical portion of genotype D when the parental connections were intact than when they were severed (**Figures 2A–E**). However, the positive effect of physiological integration on offspring growth was greater in the apical portion of genotype G when their parental connections were severed than when they were intact (**Figures 2A–E**).

**Table 2:** Statistical analysis of the effects of genotype, parental severance level, and offspring severance level on final total mass, stem mass, root mass, leaf mass, and ramet number of the apical portion of *Hydrocotyle verticillata* in the second phase.

Effect	*df*	Total mass	Stem mass	Root mass	Leaf mass	Ramet number
Genotype (G)	7, 123	0.64^ns^	0.93^ns^	0.44^ns^	0.37^ns^	0.35^ns^
Parental Severance (PS)	1, 123	0.21^ns^	0.05^ns^	<0.01^ns^	0.45^ns^	<0.01^ns^
Offspring Severance (OS)	1, 123	102.40^***^	116.93^***^	72.38^***^	69.71^**^	47.91^***^
G × PS	7, 123	2.01^#^	2.17^*^	1.97^#^	1.52^ns^	1.77^ns^
G × OS	7, 123	1.15^ns^	1.32^ns^	0.89^ns^	0.98^ns^	0.61^ns^
PS × OS	1, 123	0.68^ns^	0.64^ns^	<0.01^ns^	1.01^ns^	0.01^ns^
G × PS × OS	7, 123	1.12^ns^	0.93^ns^	1.78^ns^	1.29^ns^	1.09^ns^

The F values, degrees of freedom (df) and significance levels (*** P<0.001,** P<0.01, # 0.05<P< 0.1, * P<0.05, and ns P>0.1 ) of three-way ANOVA are given. Values are bold where P < 0.05.

**Figure 3:**
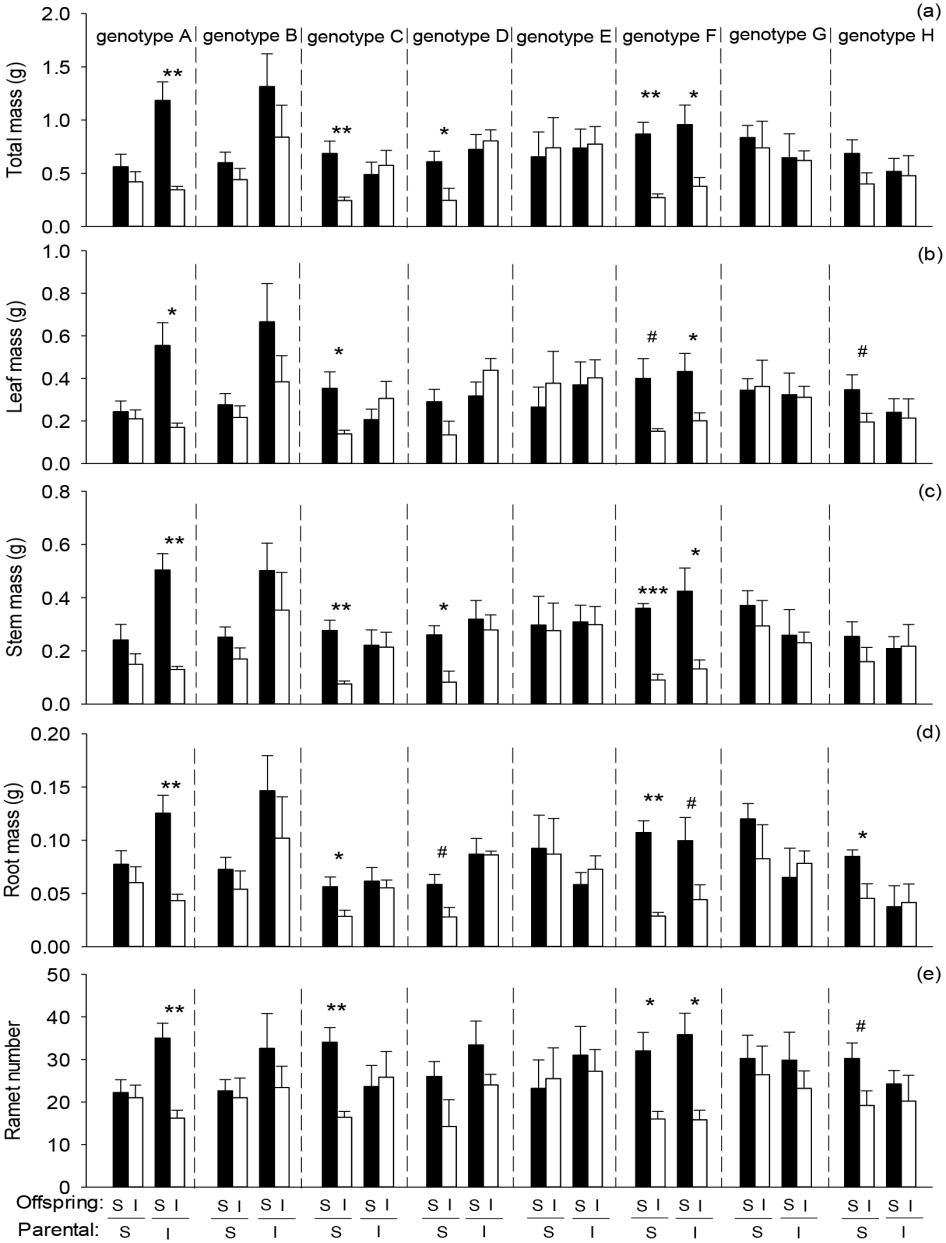
Effects of genotype, parental severance level, and offspring severance level on final **(A)** total mass, **(B)** leaf mass, **(C)** stem mass, **(D)** root mass, and **(E)** ramet number of the basal portion of *Hydrocotyle verticillata* in the second phase. S and I stand for severance and intact, respectively. Symbols indicate the levels of differences between offspring severed and intact within a genotype (***P < 0.001, **P < 0.01, *P < 0.05, and ^#^0.05 < *P* < 0.1).

Offspring severance had highly significant positive effects on total mass, leaf mass, root mass, stem mass, and ramet number of the basal portion in the second phase (**Figures 3A–E**; **Table 3**: *F* = 9.45 – 33.99; *P* = 0.000 – 0.003). Parental severance markedly decreased total mass, leaf mass, and stem mass of the basal portion in the second phase (**Figures 3A–C**; **Table 3**: *F* = 5.56 – 6.90; *P* = 0.010 – 0.020). The negative effect of physiological integration on offspring growth was greater in the basal portion of genotype A when their parental connections were intact than when they were severed (**Figures 3A–E**).

**Table 3:** Statistical analysis of the effects of genotype, parental severance level, and offspring severance level on final total mass, stem mass, root mass, leaf mass, and ramet number of the basal portion of *Hydrocotyle verticillata* in the second phase.

Effect	*df*	Total mass	Stem mass	Root mass	Leaf mass	Ramet number
Genotype (G)	7, 123	0.95^ns^	1.12^ns^	1.25^ns^	0.73^ns^	0.23^ns^
Parental Severance (PS)	1, 123	5.90^*^	6.90^*^	2.15 ^ns^	5.56^*^	1.75 ^ns^
Offspring Severance (OS)	1, 123	20.67^***^	33.99^***^	20.36^***^	9.45^**^	22.11^***^
G × PS	7, 123	1.49^ns^	1.42^ns^	1.86^#^	1.50^ns^	0.92^ns^
G × OS	7, 123	1.58^ns^	1.85^#^	1.46^ns^	1.50^ns^	1.27^ns^
PS × OS	1, 123	2.24^ns^	3.21^#^	3.03^#^	1.06^ns^	0.01^ns^
G × PS × OS	7, 123	1.61^ns^	1.44^ns^	0.94^ns^	1.78^ns^	1.28^ns^

The F values, degrees of freedom (df) and significance levels (*** P<0.001,** P<0.01, # 0.05<P< 0.1, * P<0.05, and ns P>0.1 ) of three-way ANOVA are given. Values are bold where P < 0.05.

**Figure 4:**
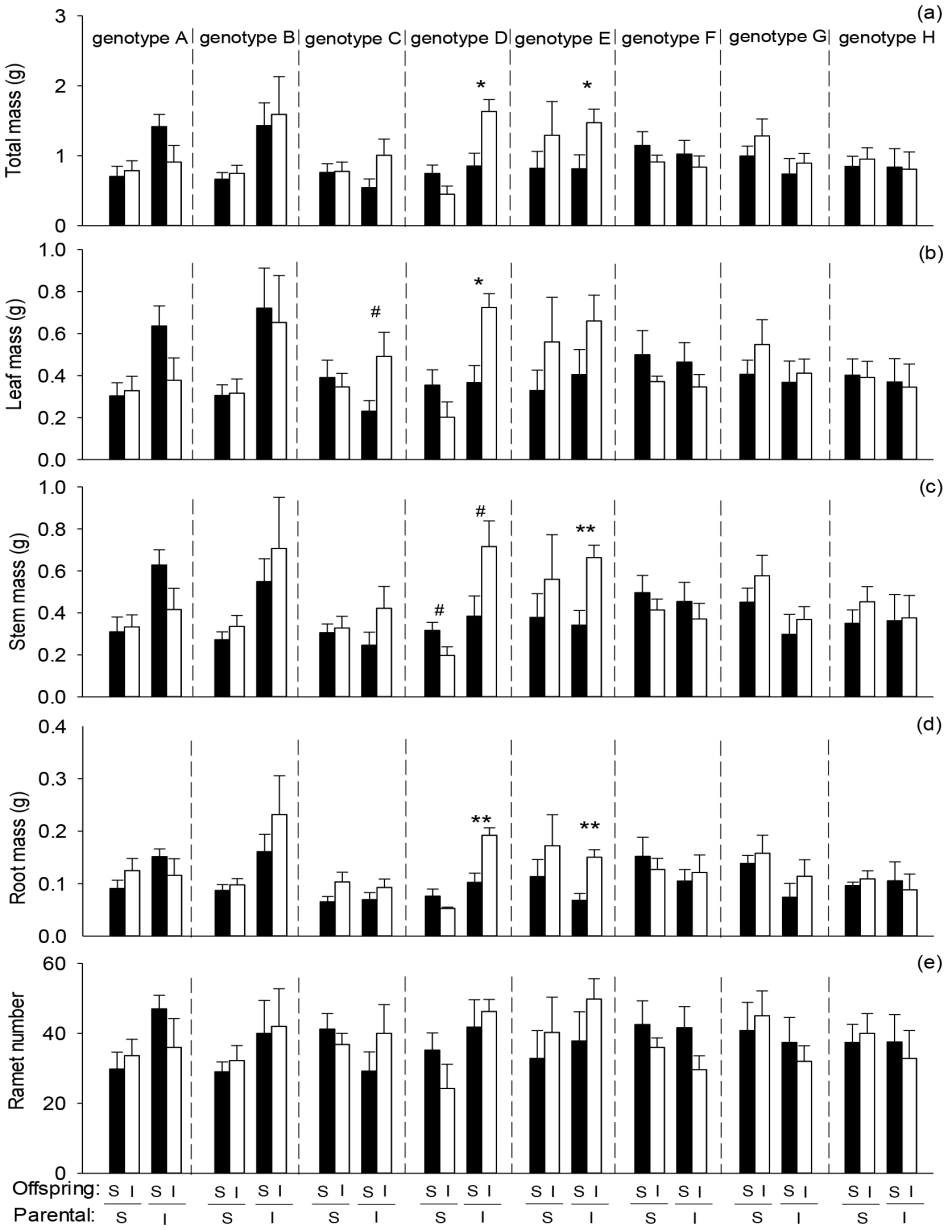
Effects of genotype, parental severance level, and offspring severance level on final **(A)** total mass, **(B)** leaf mass, **(C)** stem mass, **(D)** root mass, and **(E)** ramet number of the whole clone of *Hydrocotyle verticillata* in the second phase. S and I stand for severance and intact, respectively. Symbols indicate the levels of differences between offspring severed and intact within a genotype (**P < 0.01, *P < 0.05, and ^#^0.05 < *P* < 0.1).

Averaged across genotypes, offspring severance increased root mass of the whole clone in the second phase (**Figure 4D**; **Table 4**: *F* = 4.10; *P* = 0.045). Parental severance had little effect on offspring performance of the whole clone (**Figures 4A–E**; **Table 4**). However, the effects of parental severance on total mass, stem mass, and root mass of the whole clone varied among different genotypes in the second phase, as indicated by the significant interactive effect of parental severance × genotype (**Figures 4A, C, D**; **Table 4**: *F* = 2.39 – 3.04; *P* = 0.006 – 0.025). The positive effect of physiological integration on offspring biomass accumulation was greater in genotype D of the whole clone when the parental connections were intact than when they were severed (**Figures 4A–D**).

**Table 4:** Statistical analysis of the effects of genotype, parental severance level, and offspring severance level on final total mass, stem mass, root mass, leaf mass, and ramet number of the whole clone of *Hydrocotyle verticillata* in the second phase.

Effect	*df*	Total mass	Stem mass	Root mass	Leaf mass	Ramet number
Genotype (G)	7, 123	0.53^ns^	0.66^ns^	1.27^ns^	0.31^ns^	0.13^ns^
Parental Severance (PS)	1, 123	2.34 ^ns^	1.97^ns^	0.09 ^ns^	*3.36^#^*	0.59 ^ns^
Offspring Severance (OS)	1, 123	1.37^ns^	2.27^ns^	4.10^*^	0.37^ns^	0.01^ns^
G × PS	7, 123	2.39^*^	2.70^*^	3.04^**^	1.89^#^	1.62^ns^
G × OS	7, 123	0.99^ns^	0.90^ns^	0.86^ns^	1.11^ns^	0.77^ns^
PS × OS	1, 123	0.61^ns^	0.61^ns^	0.49^ns^	0.54^ns^	0.05^ns^
G × PS × OS	7, 123	1.20^ns^	1.21^ns^	0.98^ns^	1.34^ns^	0.82^ns^

The F values, degrees of freedom (df) and significance levels (** P<0.01, * P<0.05, # 0.05<P< 0.1, and ns P>0.1) of three-way ANOVA are given. Values are bold where P < 0.05.

## 4 Discussion

In the first phase, the negative effects of severance on the apical portion balanced the positive effects of severance on the basal portion, resulting in no net effect of severance on biomass accumulation and ramet number of the whole clone of *H. verticillata*. These results are consistent with many previous studies, which showed that severance can reduce the growth of recipient (i.e., offspring or younger) ramets and improve the growth of donor (i.e., parent or older) ramets (Wang et al., 2009; Roiloa et al., 2010; Xiao et al., 2011; You et al., 2013). When the growth reduction in recipient ramets equals the growth increase in donor ramets, physiological integration does not affect the growth of the whole clone (Wang et al., 2014). Furthermore, some differences in the effects of physiological integration were detected between the genotypes of *H. verticillata* (**Figure 1**), which was primarily due to differences in the capacity for resource sharing (Alpert et al., 2003; Si et al., 2020).

The results partly supported the first hypothesis that parental severance can affect offspring performance of *H. verticillata.* Parental severance decreased total mass, leaf mass, and stem mass of the basal portion, but had little effect on offspring performance of the apical portion and the whole clone in the second phase. Because the initial ramets of the basal portion in the second phase were directly derived from the first phase, the effects of parental severance may be stronger on the basal portion. Previous studies have indicated that the size and biomass of offspring ramets typically decrease with increasing vegetative generation (Wang et al., 2014; Zhang et al., 2022b). Thus, the ability for resource translocation may weaken from the basal portion to the apical portion and then have little effect on the whole clone in the second phase.

Parental severance had varying effects on offspring performance of the eight *H. verticillata* genotypes. For example, compared with parental severance, intact connections increased biomass accumulation of genotypes A, B, and D but either decreased or had little effect on the other genotypes in the basal portion, the apical portion and the whole clone of *H. verticillata* in the second phase (**Figures 2**–**4**). Therefore, the results supported the second hypothesis that parental severance on offspring performance can vary between the genotypes. One plausible explanation is the variation in the capacity for physiological integration between different genotypes (Si et al., 2020). Furthermore, intact connections increased biomass per ramet of genotypes A, B, and D of the apical portion in the first phase, therefore, ramet size may play an important role in offspring growth. Larger ramets may have a greater capacity for resource provisioning than smaller ramets do. The promoted growth of the offspring ramets is likely due to the importation of carbohydrates, nutrients, and/or water translocated from their larger mother ramets. Our findings are consistent with those of previous studies, which reported that larger offspring produced in favorable parental environments benefit subsequent offspring growth (Dong et al., 2019; Portela et al., 2020), as stated by the ‘silver-spoon’ effect, in which parent plants in favorable conditions can provide more resources to their offspring (Roach and Wulff, 1987; Grafen, 1988). Such advantages can promote plant growth and may in turn increase intraspecific competitiveness and invasiveness, further influencing competition and population dynamics in natural environments.

Although there was no significant interaction effect of parental severance × offspring severance × genotype on offspring growth in *H. verticillata*, the positive effects of physiological integration on offspring biomass accumulation and ramet number were greater for genotype D of the apical portion and the whole clone when their parental connections were intact than when they were severed, whereas the positive effect in the apical portion was greater for genotype G when their parental connections were severed than when they were intact. Therefore, our results do not support the third hypothesis that the positive effect of physiological integration on offspring growth is greater when parental connections are intact. The results suggested that parental effects can influence the capacity for physiological integration of offspring generations and that the effects may vary among genotypes. Our findings do not support another potential benefit of parental effects, which state that offspring generations could gain an advantage when establishing under the same or similar conditions to those experienced by their parents (Dong et al., 2018; Portela et al., 2020). In this study, physiological integration had little effect on the whole clone of *H. verticillata* in the first phase. Previous studies have shown that physiological integration significantly increased the growth of the whole clone of some species (Zhou et al., 2017; Estrada et al., 2020; Wang et al., 2021a); in that case, a greater advantage in parental generations may be transferred to offspring generations. However, few studies focus on parental effects of physiological integration in clonal plants (Adomako et al., 2021), and the underlying mechanisms require further research.

## 5 Conclusions

We conclude that parental effects of physiological integration may not always enhance offspring growth of clonal plants. Our results also suggest that clonal parental effects of physiological integration can influence the capacity for physiological integration of offspring generations, and the effects may vary among different genotypes. Thus, parental effects of physiological integration may further influence intraspecific competition and population dynamics. Our study highlights the importance of parental effects of physiological integration in shaping clonal plant growth.

## Data Availability

The raw data supporting the conclusions of this article will be made available by the authors, without undue reservation.
